# Plasmid and chromosomal sequences of *Pantoea agglomerans* isolated from air in Fort Collins, Colorado

**DOI:** 10.1128/mra.00341-25

**Published:** 2025-06-09

**Authors:** Ashley Freedman, Kendall Malmstrom, Paige Gruber, Bradley R. Borlee, Carolina Mehaffy

**Affiliations:** 1 Department of Microbiology, Immunology, and Pathology, Colorado State University 164597 , Fort Collins, Colorado, USA; Montana State University, Bozeman, Montana, USA

**Keywords:** airbiome, whole genome sequencing, *Pantoea*, drug resistance, plasmid

## Abstract

A *Pantoea agglomerans* isolate collected from an air sample was sequenced using Nanopore technology. The genome contains five contigs, totaling 4,850,529 bp with 55.14% GC content and including two chromosomal DNA contigs and three circular plasmids. In total, the genome contains 5,061 annotated genes, including 12 associated with drug resistance.

## ANNOUNCEMENT

An air sample collected near the Dixon reservoir in Fort Collins, Colorado, USA (40°33′05.6″N, 105°08′32.0″W) by an Ultrasonic Personal Air Sampler (1) for 45 min and a flow rate of 2 L/min was plated on Tryptic Soy Agar (TSA) and incubated at room temperature for 24–48 h. A yellow-pigmented colony was obtained and streaked for isolation on TSA. Glycerol stocks of the isolate AF_air_401 were prepared and stored at −80°C. The isolate was identified as *Pantoea agglomerans* by matrix-assisted laser desorption/ionization time-of-flight (MALDI-TOF) (score = 2.41) (Bruker).

*P. agglomerans* is an opportunistic, rod-shaped Gram-negative bacterium (2). *Pantoea* species have applications in biological industries (3, 4). AF_air_401 isolate was sequenced as *P. agglomerans,* species are potential carriers of antibiotic resistance genes (ARGs) (5).

The isolate was inoculated in two 5 mL of LB media and grown with shaking at room temperature for 48 h. Biomass was harvested, and genomic DNA (gDNA) was isolated using the Monarch Genomic DNA Purification Kit (#T3010S, NEB) following the manufacturer’s instructions for Gram-negative isolates. Oxford Nanopore Technologies Rapid PCR Barcoding Kit 24 V14 (SQK-RPB114.24) was used for library preparation following the manufacturer’s protocol, except PCR cycles were increased to 25. gDNA was not sheared or size-selected. gDNA was quantified by Qubit (dsDNA HS Assay Kit [Q32851, ThermoFisher]). Mk1B device (MN45679) and MinION Flow Cell R10 Version (FLO-MIN114, ID FAZ80926) were used for sequencing. MinKnow v24.06.8 with default parameters and Basecalling fast model v4.3.0 with parameters: 400 bps and MinQ score 8 were used for WGS and post-run trimming.

Assembly and polishing were performed in the Galaxy server v24.1.4.dev0 (6–8). Visualization and annotation were performed in Proksee (9). All tools were configured using default parameters. Raw reads (327,922 reads [N50 = 3,990]) were pre-processed in UseGalaxy.eu using Necat v0.0.1_update20200803+galaxy0 (10) with an estimated genome size of 5 Mb. All other tools were accessed in UseGalaxy.org. Reads were concatenated by the Concatenate Multiple Data sets v.0.2 tool (11). Assembly and circularization were performed using Flye v2.9.5+galaxy0 (12, 13), resulting in five contigs (Table 1). Quast v5.2.0+galaxy1 determined assembly statistics (14–17) (Table 1). The assembled genome was polished with workflow “Assembly polishing with long reads v.01,” which utilizes Racon v1.5.0+galaxy1 (18) and minimap2 v2.28+galaxy1 (19–21). Circular assemblies were not rotated. N50 after polishing was 3,486,650 bp (GC = 55.14%). Prokka v1.14.6 (22, 23) and CARD RGI v6.0.3 (24) were used to annotate the genome. The 16S rDNA sequence was compared against the 16S rDNA bacteria and archaea database using BLAST (25), confirming the isolate as *Pantoea agglomerans* (99.21% identity, 98% query).

**TABLE 1:** Assembly information and list of identified Antimicrobial Resistant Genes (ARGs) per contig

Contig^*a*^	Length (bp)	Coverage (%)	Circular (bp)	Number of annotated CDS	Antibiotic resistance genes	Antibiotic class resistance
Contig_1	3,486,465	59	N	4,285	emrR (efflux pump) kpnH (efflux pump) rsmA (efflux pump) EF-Tu mutation Crp (efflux pump) uhpT mutation pbp3 (transpeptidase) adeF (efflux) × 2 msbA (efflux) kpnEF (efflux) fosA8 (inactivation)	Fluoroquinolone Multiple Multiple Elfamycin Multiple Fosfomycin Beta-lactams Multiple Nitroimidazole Multiple Fosfomycin
Contig_3	623,087	53	N			
Contig_5	528,879	48	Y	562	Not identified	N/A^*b*^
Contig_6	182,235	57	Y	177	arnT (target alteration)	Peptide antibiotic
Contig_4	29,860	91	Y	37	Not identified	N/A

^*a*^ Contigs 1 and 3 make the chromosomal DNA of *P. agglomerans* isolated in this study.

^*b*^ N/A: Not applicable.

**Fig 1:**
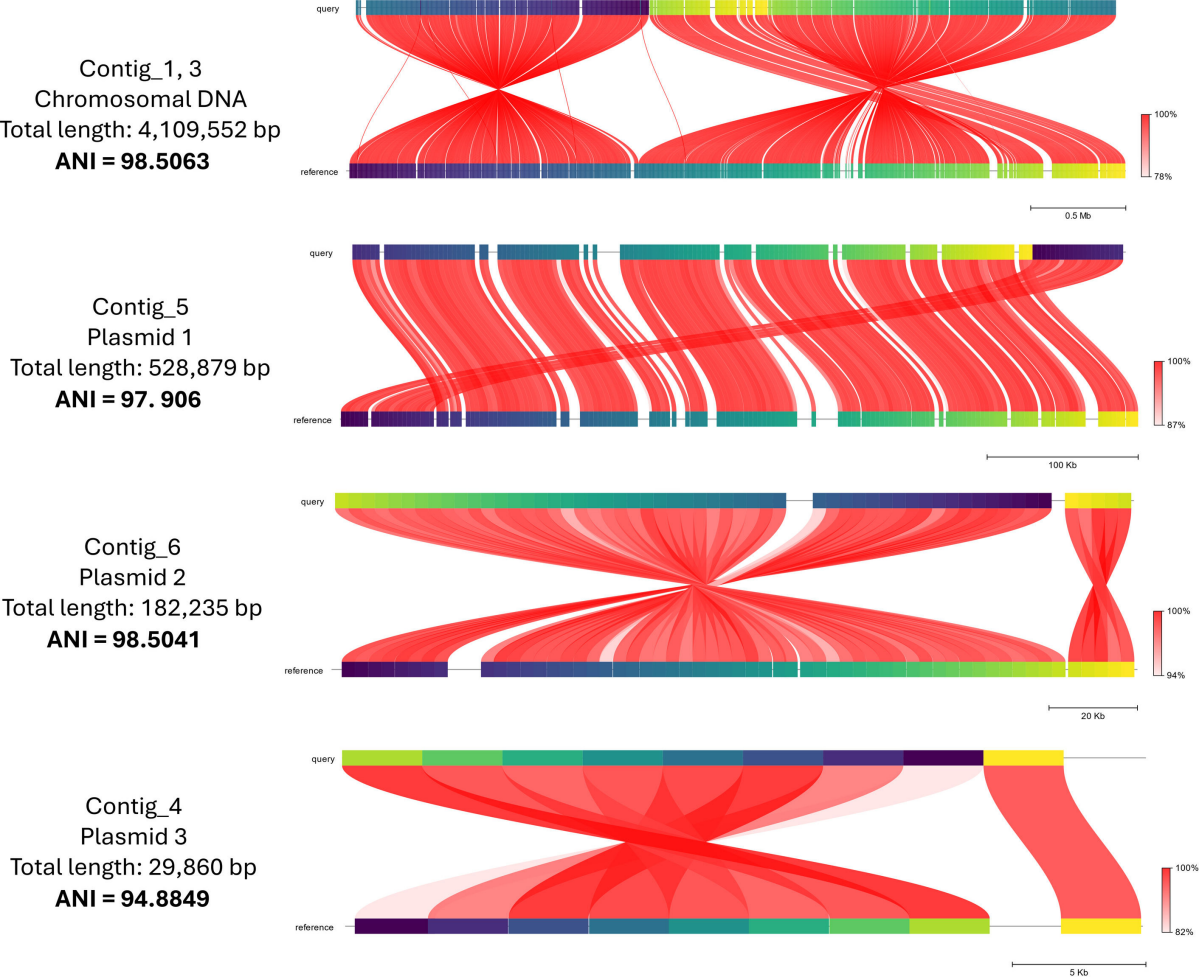
Results from FastANI (average nucleotide identity) against the *P. agglomerans* reference genomes (accession: CP077366, CP077368, CP077367, and CP155069 from top to bottom). Each red line segment denotes a reciprocal mapping between the query (top) and reference (bottom) genomes. Shade of red represents the ANI percentage. Colors on the query and reference genome (horizontal segments) represent orthologous fragments of 3,000 bp.

Multiple ARGs were identified (Table 1); many were located on the chromosome (contigs 1 and 3), reducing the possibility of horizontal gene transfer. FastANI v1.1.0 (26) was used to compare the assembled genome with the reference *P. agglomerans* genome (NCBI accession = ASM1904838v1), containing a chromosome and two plasmids. Average nucleotide identity (ANI) for all three comparisons was greater than 97% (Fig. 1). The smallest plasmid sequence had a 95.91% identity (93% query) to a *Pantoea* sp. BRR-3P plasmid via BLAST (25) and an ANI of 94.9% (Fig. 1), indicating a potentially uncharacterized plasmid.

## Data Availability

This whole genome assembly has been deposited in GenBank with accession number JBMHFE000000000 under biosample SAMN47517789. The version described in this paper is the first version. Raw reads were submitted to the NCBI Sequence Read Archive with accession number SRR32812906.
